# Expression of hypoxia inducible factor 1α and 2α and its association with vitamin C level in thyroid lesions

**DOI:** 10.1186/s12929-017-0388-y

**Published:** 2017-10-30

**Authors:** Paweł Jóźwiak, Piotr Ciesielski, Agnieszka Zaczek, Anna Lipińska, Lech Pomorski, Marek Wieczorek, Magdalena Bryś, Ewa Forma, Anna Krześlak

**Affiliations:** 1 Department of Cytobiochemistry, Faculty of Biology and Environmental Protection, University of Lodz, Pomorska 141/143, 90-236, Lodz, Poland; 2 Department of General and Oncological Surgery, Medical University of Lodz, Pomorska 251, 92-213 Lodz, Poland; 3 Department of Neurobiology, Faculty of Biology and Environmental Protection, University of Lodz, Pomorska 141/143, 90-236, Lodz, Poland

**Keywords:** HIF-1α/ HIF-2α/ vitamin C/GLUT1/ thyroid cancer

## Abstract

**Background:**

Cells adapt to hypoxia by transcriptional induction of genes that participate in regulation of angiogenesis, glucose metabolism and cell proliferation. The primary factors mediating cell response to low oxygen tension are hypoxia inducible factors (HIFs), oxygen-dependent transcription activators. The stability and activity of the α subunits of HIFs are controlled by hydroxylation reactions that require ascorbate as a cofactor. Therefore, deficiency of intracellular vitamin C could contribute to HIFs overactivation. In this study, we investigated whether vitamin C content of human thyroid lesions is associated with HIF-1α and HIF-2α protein levels.

**Methods:**

Expression of HIF-1α and HIF-2α as well as vitamin C content was analyzed in thyroid lesions and cultured thyroid carcinoma cell lines (FTC-133 and 8305c) treated with hypoxia-mimetic agent (cobalt chloride) and ascorbic acid. The expression of HIFs and hypoxia–induced glucose transporters were determined by Western blots while quantitative real-time PCR (qRT-PCR) was performed to detect HIFs mRNA levels. Ascorbate and dehydroascorbate levels were measured by HPLC method.

**Results:**

We found an inverse correlation between vitamin C level and HIF-1α but not HIF-2α expression in thyroid lesions. These results agree with our in vitro study showing that vitamin C induced a dose - dependent decrease of HIF-1α but not HIF-2α protein level in thyroid cancer cells FTC-133 and 8305C. The decreased HIF-1α expression was correlated with reduced expression of hypoxia-related glucose transporter 1 (GLUT1) in thyroid cancer cells.

**Conclusion:**

The results demonstrate that HIF-1α activation is associated with vitamin C content in thyroid lesions. Our study suggests that high tumor tissue ascorbate level could limit the expression of HIF-1α and its targets in thyroid lesions.

## Background

Rapidly proliferating cancer cells require adaptive metabolic changes allowing them to continue biosynthesis and growth under conditions of hypoxia and low nutrient availability [1, 2]. The capacity of living cells for adaptation to hypoxia is mediated, in part, by hypoxia-inducible transcription factors (HIFs). HIFs form heterodimers composed of oxygen labile HIF-α subunit and constitutively expressed HIF-β subunit. Three related α isoforms (HIF-1α, HIF-2α and HIF-3α) and β-subunit are members of the basic helix-loop-helix (bHLH) proteins of the PER-ARNT-SIM (PAS) family of transcription regulators [3]. HIF transcriptional response to lack of oxygen has been mostly attributed to HIF-1α and HIF-2α. Under hypoxia conditions, HIF-α subunits enter the nucleus, where they dimerize with HIF-1β and then bind to a hypoxia response element (HRE) within the promoter and enhancer regions of the target genes [4, 5]. HIF-1α preferentially drives the transcription of genes that control glycolysis, angiogenesis and glucose transport pathways, while HIF-2α is involved in the regulation of genes important for tumor growth and cell cycle progression [6–8]. Increased HIFs activity has been shown to promote tumor progression and resistance to chemo- and radiotherapy and is associated with poor patient prognosis [9, 10]. Therefore, there is a great interest in potential inhibitors of HIFs for use in cancer therapy.

Activity and stability of HIFs are controlled by posttranslational modification of the α subunits mediated by specific prolyl hydroxylases (PHDs). Proline hydroxylation is a signal for the recruitment of ubiquitin ligase complex that leads to polyubiquitynation and rapid proteosomal degradation of HIF-α subunit. Thus, optimal PHDs activity directly suppress HIFs-dependent transcriptional regulation. These enzymes have a non-heme iron in the catalytic site and their activities depend on the supply of substrates (oxygen and 2-oxoglutarate) and a cofactor (ascorbate) that stabilizes the active-site Fe in the reduced state [11]. Although ascorbate is a specific reducer of PHDs and its deficiency limits HIF hydroxylases activity, this cofactor has received relatively little attention as a regulator of HIFs [12, 13].

The role of vitamin C in cancer has been investigated for a long time but the results of the studies are conflicting. Howerver, more recent studies have shown that ascorbate administration can significantly reduce tumor growth rate in mice [14]. Clinical trials demonstrated that millimolar concentration of vitamin C in plasma could be achieved with intravenous infusion and may have therapeutic effect or can enhance chemotherapy efficacy [15, 16]. Most cells accumulate ascorbate (AA) to low millimolar levels by active transport from the plasma via Na^+^ − dependent vitamin C transporters (SVCTs) or dehydroascorbic acid (DHAA) absorption via Na^+^ −independent facilitative glucose transporters (GLUTs) followed by intracellular reduction [17, 18]. In physiological conditions the DHAA level in serum is very low but it is suggested that GLUT transporters may have significant impact on intracellular level of vitamin C in regions of inflammation associated with tumor where AA may be oxidized to DHAA [19, 20]. Mammalian facilitative glucose transporters GLUT1 and GLUT3 mediate DHAA transport with a similar efficiency to that of glucose and expression of these two carriers is controlled by hypoxia-inducible transcription factors [21]. However, the relationship between AA and DHAA levels and HIF expression patterns in human tumors has been poorly investigated. Therefore, the aim of this study was to estimate HIF-1α and HIF-2α expression and determine whether the HIFs level in human thyroid lesions is associated with the ascorbate and dehydroascorbate content.

## Methods

### Patients and samples

The analyzed specimens were obtained from the Department of General and Oncological Surgery of the Medical University of Łódź. Samples of thyroid lesions were obtained from 106 patients who underwent surgical resection due to nodular thyroid diseases. The tissue specimens collected in the operation room were prepared and evaluated by an experienced pathologist. Thyroid specimens were immediately frozen after resection and stored at −80 °C until needed. Clinicopathological characteristics of specimens are shown in Table 1. Typing and staging of tumors were carried out according to the system accepted by the International Union Against Cancer (UICC, 2010). The studies were performed with the approval of the Bioethical Commission of the Medical University of Lodz (RNN/493/13/KB).

**Table 1:** Characteristics of patients and surgically resected thyroid lesions

Characteristic	Number of patients
Sample size	106
Median age (range) 56 (30–74)	
Gender	
Female	87
Male	19
Papillary carcinoma	35
Stage	
I	8
II + III	13
IV	14
Lymph node metastasis	
Yes	17
No	18
Follicular carcinoma	7
Follicular adenoma	13
Nodular goiter	51

### Cell cultures and treatment

The thyroid cancer cells lines FTC133 (follicular thyroid cancer cells) and 8305c (anaplastic thyroid cancer cells) were obtained from the European Collection of Cell Cultures (ECACC), (Wiltshire, UK). Cells were maintained in DMEM supplemented with 2 mM glutamine and 10% fetal bovine serum (FBS) in a humidified atmosphere containing 5% CO_2_ at 37 °C. Cells were treated with 0.1–1 mM L-ascorbic acid. Dithiothreitol (DTT) was added to culture media in final concentration of 100 μM to stabilize vitamin C in ascorbate form. To mimic hypoxia conditions, hypoxia agent cobalt chloride (CoCl_2_) was added to the medium in final concentration of 100 μM.

### RNA isolation and cDNA synthesis

Total RNA was isolated using Fenozol reagent (A&A Biotechnology, Gdynia, Poland). The RNA quality was confirmed by electrophoresis on an agarose gel and spectrophotometrically. RNA samples with a 260/280 nm ratio in the range of 1.8–2.0 were used for further analysis. cDNA was synthesized from 2 μg of total RNA using a RevertAid™ First-Strand cDNA Synthesis Kit (Thermo Fisher Scientific Inc., Waltham, MA, U.S.A.), following the manufacturer’s instructions.

### Quantitative real-time PCR

Quantitative real-time PCR with commercially available primers and fluorescent probes (TaqMan^®^ Gene Expression Assay; Applied Biosystems™, Foster City, CA, USA) was used to detect the expression of *HIF1A, HIF2A*, *SLC2A1* and *SLC2A3* genes (coding for HIF-1α, HIF-2α, GLUT1 and GLUT3 respectively) in different types of thyroid lesions. The assay numbers for studied genes were as follows: Hs00153153_m1, Hs01026149_m1, Hs00892681_m1, Hs00359840_m1 and Hs99999905_m1 (reference gene: GAPDH).

The RT-qPCR reaction was carried out using the Mastercycler ep realplex (Eppendorf) as previously described [22]. The 2^ΔCt (Ctgene–Ct*GAPDH*)^ method was used to calculate the expression levels of studied genes. The 2^-ΔCt^ values were re-calculated into relative copy number values (number of HIF-1α or HIF-2α mRNA copies per 1.000 copies of GAPDH mRNA).

### Protein extraction

Thyroid specimens were homogenized using a Potter’s homogenizer in 10 volumes of ice-cold RIPA buffer (50 mM Tris-HCl – 150 mM NaCl – 1% Triton X-100 – 0.5% DOC – 0.1% SDS – 2 mM EDTA_,_ pH 7.4) with 1 mM phenylmethylsulfonyl fluoride (PMSF) to inhibit protease activity. Cell pellets were solubilized for 30 min on ice in RIPA buffer and then sonicated using Vibra Cell ™ (Sonics) sonicator. The efficiency of homogenization was monitored by phase-contrast light microscopy. The supernatants obtained after centrifugation of the tissue and cell lysates at 10000 x g at 4 °C for 10 min were collected and saved for further analysis.

### Western blot analysis

Protein samples (40 μg protein/lane) were resolved by 8% SDS-PAGE and electrotransferred onto Immobilon-P transfer membranes (Millipore, Bedford, MA, USA). In order to verify the quality of transfer Ponceau S staining was used prior to blocking the membrane. The blots were incubated for 2 h at room temperature with the rabbit anti-human HIF-1α polyclonal antibodies in a 1:400 dilution (Santa Cruz Biotechnology^®^ Inc., Santa Cruz, CA, USA), mouse anti-human HIF-2α monoclonal antibodies in a 1:500 dilution (Santa Cruz Biotechnology^®^ Inc.), rabbit anti-human GLUT1 polyclonal antibodies in a 1:1000 dilution (Abcam^®^ Cambridge, UK) or mouse anti-human GLUT3 monoclonal antibodies in a 1:500 dilution (Santa Cruz Biotechnology^®^ Inc.). After washing with TBS-T (0.1% Tween-20 in Tris-buffered saline, TBS) the blots were incubated with horseradish peroxidase-labeled goat anti-mouse or anti-rabbit IgG antibodies (Santa Cruz Biotechnology^®^ Inc.). The proteins were visualized on X-ray film by the chemiluminescence method. Gel-Pro Analyzer software version 3.0 (Media Cybernetics Inc., Bethesda, MD, USA) was used for densitometry analysis of the protein bands on blots or gels. To confirm that the same amounts of proteins were loaded into each lane, the standard silver staining method was used for total protein identification on the gels or blots were re-probed with anti-β-actin antibody following a stripping protocol. In case of tissue proteins analyses, during the preliminary Western blot experiment two samples with mean expression of studied proteins were chosen (one for HIF-1α and one for HIF-2α) and then these samples were applied as a reference samples on each gel. Integrated optical density (IOD) of total proteins after silver staining was divided by IOD of total proteins of reference sample. For further analysis only samples with similar IOD of total proteins were used. The level of HIF-1α and HIF-2α expression in thyroid specimens was normalized by the reference sample (patient number 54 and 84, respectively) resolved by electrophoresis that made possible comparison of intensity of the bands from different membranes. The results of HIF proteins expression analyses are shown as a relative protein level which is a ratio of IOD of bands corresponding to HIF-1α or HIF-2α in each sample and IOD of HIFs in the reference samples.

### Ascorbate and dehydroascorbate content

Tissue samples or cell pellets were homogenized in ice cold 5% *meta-*phosphoric acid (MPA) containing 1 mM EDTA. Ten microliters of MPA buffer was used per one milligram of wet tissue. Cell suspensions (10^6^ cells/ml) were added to an equal volume of 10% cold MPA containing 2 mM EDTA. The formed precipitates were spun down by centrifugation (16.000 x g, 2 min) and then supernatants were transferred to new vials.

Ascorbate and dehydroascorabate levels were determined as previously described [23] by high-performance liquid chromatography using modified Lykkesfeld’s subtraction method with Tris[2-carboxyethyl]phosphine (TCEP) as a reducing agent [24].

#### Statistical analysis

Statistical evaluation was performed using STATISTICA version 12.0 (StatSoft Inc. 2014), data analysis software system, www. statsoft.com). The non-parametric Mann-Whitney U test was used when two groups were compared. Comparisons between more than two groups were done using Kruskal-Wallis test. For pairwise multiple comparisons Dunn’s post hoc test was used. Spearman correlation coefficient was calculated for correlation analysis. The Student’s paired t-test was used to compare the differences between treated and untreated cells. A *p* value of <0.05 was considered significant.

## Results

### Expression of HIF-1α and HIF-2α in thyroid lesions and their association with clinicopathological characteristics

**Fig. 1:**
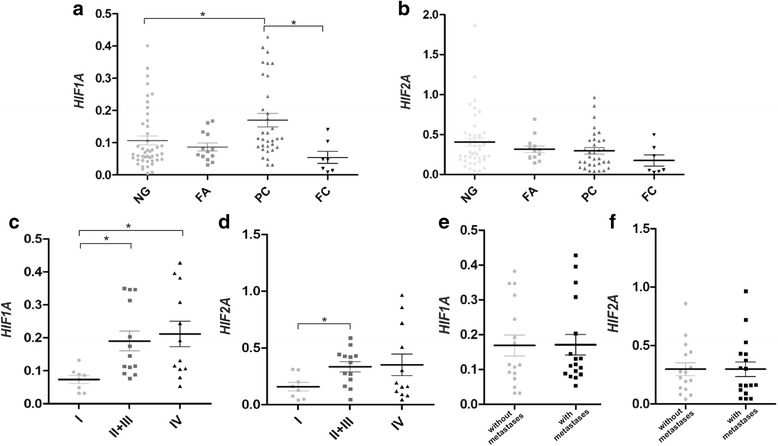
Expression of *HIF1A* and *HIF2A* mRNA in thyroid lesions. The 2^ΔCt (Ctgene–CtGAPDH)^ method was used to estimate the relative gene expression levels in the analyzed samples. The 2^-ΔCt^ values were re-calculated into relative copy number values (number of HIF-1α or HIF-2α mRNA copies per 1.000 copies of GAPDH mRNA). a and b- relative mRNA expression of *HIF1A* (**a**) and *HIF2A* (**b**) in samples of nodular goiter (NG; *n* = 45), follicular adenoma (FA; *n* = 13), papillary carcinoma (PC; *n* = 33) and follicular carcinoma (FC; *n* = 7); **c** and **d** –expression of *HIF1A* and *HIF2A* in papillary carcinoma samples characterized by different tumor stages (I *n* = 8; II + III *n* = 13; IV *n* = 12); **e** and **f** - papillary carcinoma samples of different lymph node metastasis status (with metastases *n* = 17; without metastases; *n* = 16). Data represent the means ± SEM, * *p* < 0.05

mRNA levels of HIF-1α and HIF-2α were analyzed by real-time PCR in a sample set containing non-neoplastic lesions (nodular goiters, NG), follicular adenomas (FA), papillary carcinomas (PC) and follicular carcinomas (FC). The results of *HIF1A* and *HIF2A* gene expression analysis in different types of thyroid lesions are graphed in Fig. 1. The results indicated that *HIF1A* gene expression was significantly higher in PCs than in non-neoplastic NG lesions (*p* < 0.05) (Fig. 1a). A lower abundance of HIF-1α mRNA was observed in FCs in comparison with PC cases (p < 0.05). There were no statistically significant differences in HIF-2α mRNA levels between different thyroid lesions (Fig. 1b). In the PC group, *HIF1A* and *HIF2A* expression were higher in tissue samples of patients with more advanced disease stages (Fig. 1c and d). No significant differences were noted in the expression of both genes between PC cases with and without metastases to lymph nodes (Fig. 1e and f).

**Fig. 2:**
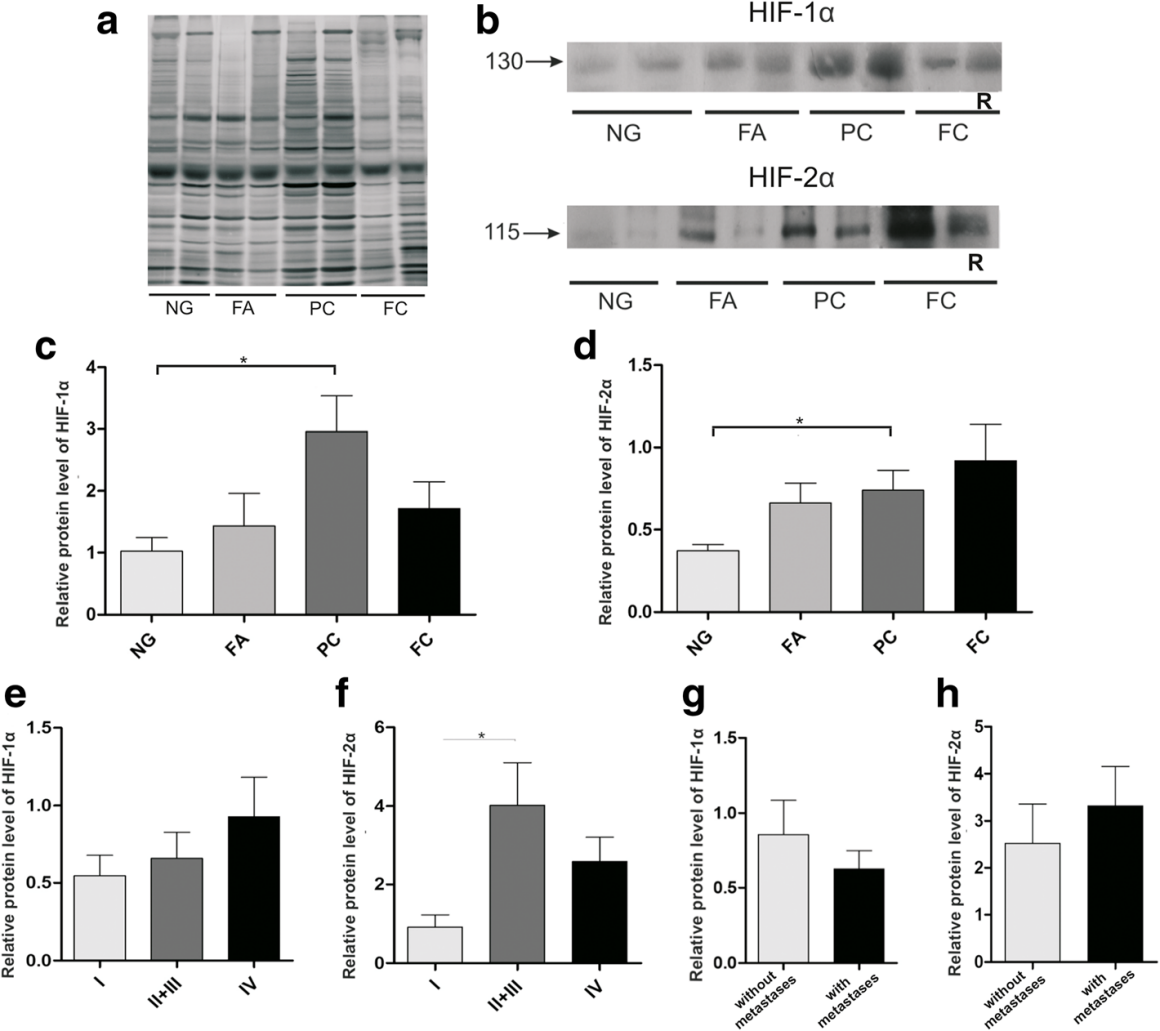
Expression of HIF-1α and HIF-2α proteins in thyroid lesions. Samples containing 40 μg of proteins were resolved by SDS-PAGE and stained with silver method to check their quality and total protein content or transferred onto membranes for immunodetection. Representative silver stained samples and Western blots of HIF-1α and HIF-2α detection in thyroid lesions are shown in **a** and **b**, respectively; nodular goiter (NG; *n* = 44), follicular adenoma (FA; *n* = 13), papillary carcinoma (PC; *n* = 33) and follicular carcinoma (FC; *n* = 7). The intensity of bands corresponding to HIF-1α and HIF-2α was analyzed by densitometry and integrated optical density (IOD) was normalized by protein content and reference sample (see Methods for details). Graphs represent mean IODs of bands from all analyzed samples. The results are presented as a mean ± SEM for HIF-1α (**c**) and for HIF-2α (**d**). **e** and **f** –expression of HIF-1α and HIF-2α, respectively in papillary carcinoma samples characterized by different tumor stages (I *n* = 8; II + III *n* = 13; IV *n* = 12); **g** and **h** - expression of HIF-1α and HIF-2α, respectively, in papillary carcinoma samples characterized by lymph node metastasis status (with metastases, *n* = 17; without metastases, *n* = 16). * *p* < 0.05; R - reference sample

To determine expression of HIF-1α and HIF-2α proteins in the thyroid lesions, homogenized tissues were examined by Western blotting. The samples of thyroid lesions homogenates were resolved by SDS-PAGE. To confirm that the same amounts of proteins were loaded into each lane, the standard silver staining method was used for total protein identification on the gels (Fig. 2a). The representative blots after HIF-1α and HIF-2α immunodetection are shown in Fig. 2b. HIF-1α and HIF-2α protein expression was detected in all types of thyroid lesions. Densitometric analysis of the bands corresponding to HIF-1α and HIF-2α (Fig. 2c and d, respectively) from all samples studied showed significantly higher immunoreactivity in papillary cancers in comparison with non-neoplastic cases (NG). No statistically significant differences were noted in the HIF-1α and HIF-2α protein levels between non-neoplastic lesions and the benign neoplasm cases (Fig. 2c and d, respectively).

A tendency towards an increased expression of HIF-1α was observed in the PC group with an increased tumor stage, although this was not statistically significant (Fig. 2e). Results also showed significantly higher HIF-2α protein expression in papillary carcinomas of stage II/III compared with less advanced tumors (stage I) (Fig. 2f). There were no significant differences in HIF-1α and HIF-2α expression between papillary carcinomas with or without metastases to lymph nodes (Fig. 2g and h, respectively). Due to the low number of FC cases, HIFs expression data were not analyzed via clinicopathological features.

**Fig. 3:**
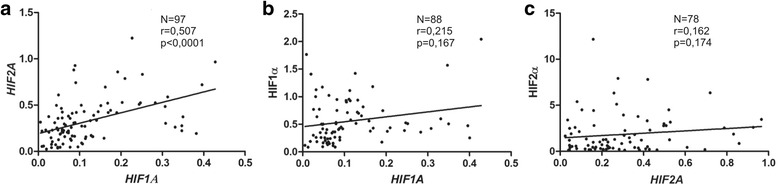
Spearman’s correlation between *HIF1A* and *HIF2A* mRNA expressions (**a**), *HIF1A* mRNA and protein level (**b**); *HIF2A* mRNA and protein expression (**c**)

The Spearman’s analysis revealed a positive correlation between *HIF1A* and *HIF2A* expression in thyroid lesions (*r* = 0.507, *p* < 0.0001) (Fig. 3a). As expected there was no correlation between HIF-1α or HIF-2α protein level and their mRNA expression (Fig. 3b and c). It is well known that the protein stability of HIF-α subunits is controlled by posttranslational hydroxylation. Nevertheless, we performed the correlation analysis to exclude the possibility that the level of HIF proteins depends mostly on gene expression regulation in thyroid lesions.

### Effect of vitamin C on activation of hypoxia-inducible factor 1α and 2α in thyroid cancer cells

**Fig. 4:**
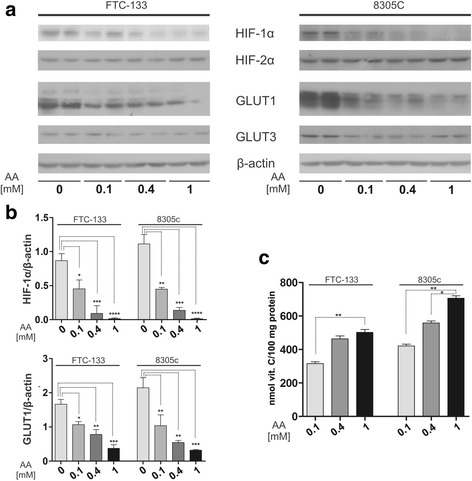
Dose-dependent effect of ascorbate on expression of HIF-1α and GLUT1. FTC-133 and 8305c cells were grown in medium containing cobalt chloride and were treated for 24 h with ascorbic acid stabilized with DTT. (**a**) Western blot analysis of HIF-1α, HIF-2α, GLUT1 and GLUT3 in thyroid cancer cells; (**b**) Results of densitometric analysis of relative HIF-1α and GLUT1 expressions in cells treated with different concentration of ascorbate; the results are shown as the mean ± SD; (**c**) Intracellular vitamin C level in FTC-133 and 8305c cells after ascorbate treatment; the results are shown as the mean ± SD. Experiments were performed in triplicate for three times; *p < 0.05, **p < 0.01, ***p < 0.001, ****p < 0.0001

As the activity of hydroxylases depends on ascorbate as a cofactor, we set out to determine whether vitamin C impacts HIF-1α and HIF-2α levels in thyroid cells. Thus, FTC-133 and 8305c cells were grown in medium containing hypoxia-mimetic agent (cobalt chloride) and were treated for 24 h with increased concentration of L-AA ranged from 0.1 to 1 mM. To prevent AA oxidation DTT was added to culture media in a final concentration of 100 μM. Results showed that in both thyroid cancer cell lines vitamin C induced a dose-dependent decrease of HIF-1α protein level. This decrease was correlated with a reduced expression of its target hypoxia associated glucose transporter GLUT1 (Fig. 4a and b). There was no impact of ascorbate on HIF-2α expression. Similarly, vitamin C did not affect the level of hypoxia-related GLUT3 glucose transporter. Quantification of vitamin C in cells 24 h after L-AA treatment was performed by HPLC-ECD using TCEP as a reducing agent according to modified method of Lykkesfeldt [24]. The results of vitamin C quantification in FTC-133 and 8305c cells after ascorbate treatment are presented in Fig. 4c. The data indicated that the dose-dependent impact of AA on HIF-1α and its target GLUT1 protein expression was associated with intracellular vitamin C content.

### Vitamin C content and its relation to hypoxia activation markers in thyroid lesions

**Fig. 5:**
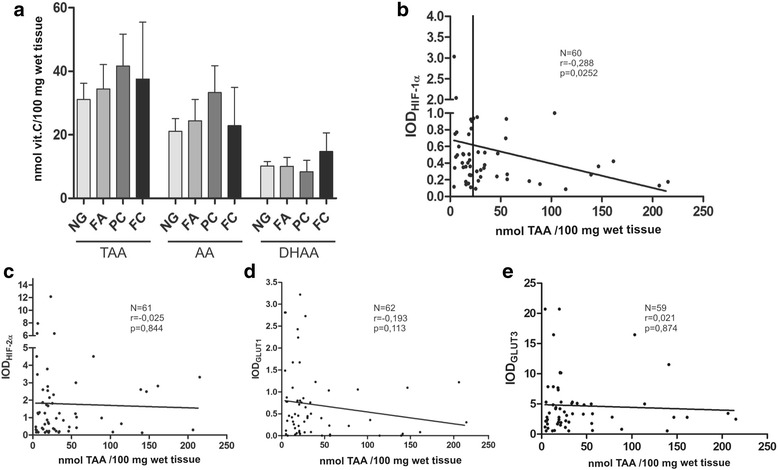
Expression of hypoxia marker HIF-1α associates with vitamin C level. (**a**) Analysis of ascorbic acid (AA), dehydroascorbic acid (DHAA) and both reduced and oxidized forms of ascorbate (TAA) levels in thyroid lesions (NG *n* = 51; FA *n* = 11; PC *n* = 23; FC *n* = 4). (b-e) Scatter plots showing HIF-1α (**b**), HIF-2α (**c**), hypoxia-related GLUT1 (**d**) and GLUT3 (**e**) proteins expression in relation to total vitamin C content in thyroid tumors (Spearman’s correlation). The median TAA value of 21,6 nmol/100 mg of tissue was used to classify sample as either ascorbate-deficient or ascorbate-replete. Ascorbate-deficient tumors had significantly higher levels of HIF-1 alpha protein than ascorbate-replete tumors (*p* < 0.01) by Mann-Whitney test

Total amount of vitamin C (TAA) as well as its reduced form (AA) were measured by HPLC. The concentration of oxidized form of ascorbate (DHAA) was calculated by subtraction of the measured AA concentration from that of TAA. Before the analyses, the stability of ascorbate in banked specimens as well as our protocol for ascorbate extraction was verified. There was no loss of ascorbate in deproteinized samples up to 12 h at room temperature and in long-term storage of intact tissue at -80 °C. The ascorbate measurements were expressed as nmoles ascorbate per 100 mg tissue. The results indicated that vitamin C was stored mainly in its reduced state in each of analyzed samples. There were no significant differences in AA and TAA levels between different types of analyzed thyroid lesions (Fig. 5a). In addition, we have observed considerable variation in the total ascorbate levels in all analyzed groups. To estimate whether vitamin C content in the thyroid relates to activation of hypoxia pathway, the total ascorbate in the samples was compared with the levels of HIF-1α, HIF-2α and hypoxia-induced glucose transporters which up-regulation in thyroid tumors we had previously reported for (Fig. 5b-d) [22]. There was a statistically significant negative correlation (*r* = − 0.288, *p* = 0.025) between ascorbate content and HIF-1α protein level. Most of specimens with high ascorbate levels had low HIF-1α expression, whereas those with highest HIF-1α immunoreactivity were ascorbate deficient (Fig. 5b). A similar trend was seen for GLUT1 protein (Fig. 5d), however the results were not statistically significant.

## Discussion

Hypoxia is a common condition found in a wide range of solid tumors and has been accepted to play an important role in cancer development and progression. Tumor adaptation to hypoxia depends mostly on HIF-1α and HIF-2α. Both isoforms are regulated by oxygen-dependent hydroxylation that results in intracellular degradation of proteins by the proteasome pathway.

HIF-1α and HIF-2α have a similar structure and function but they have unique tissue and cell type distributions [2, 3, 25]. Recent data indicate that regulation of HIF-2 target genes depends on tissue type, tumor type and co-expression with HIF-1 [26–28]. Studies concerning the role of HIFs in thyroid carcinoma are very limited. Moreover, there is no study to date which looked at both isoforms expressions in the same specimens. Therefore, in this study, we investigated whether there is any difference in the expression of HIF-1α and HIF-2α between malignant and benign neoplasms, as well as non-neoplastic thyroid lesions. Our results showed a higher intensity of bands corresponding to HIF-1α in majority of malignant carcinoma cases in comparison with nodular goiters (Fig. 2b and c). An association between HIF-1α expression and tumor type was previously noted by Burrows et al. [29] who observed significantly higher HIF-1α level in all types of thyroid malignancy, especially in follicular and anaplastic carcinomas, than in normal tissue. Another immunohistochemical study also showed higher HIF-1α immunoreactivity in most of PC cases in comparison with paired normal tissue samples [30]. Authors observed increased HIF-1α staining intensity in higher stage and metastatic cancers. Similarly, we noted a higher tendency for HIF-1α protein expression in more advanced papillary cancers, although it was not statistically significant (Fig. 2e).

HIF-1α and HIF-2α showed some overlap of target genes, however both of them have also distinct downstream targets. Therefore, hypoxia-induced metabolic reprogramming varies depending on the relation between HIF-1α and HIF-2α expressions. There is little information in the literature regarding the HIF-2α expression in thyroid cancer. In this study, we observed significantly higher HIF-2α immunoreactivity in the PC group than in non-neoplastic thyroid lesions (Fig. 2b and d). The expression of HIF-2α protein was elevated in PC cases with higher tumor stage (Fig. 2d). Our results agree with the previously reported data by Wang et al. [31] who showed statistically significant differences in HIF-2α protein expression levels between PCs and normal thyroid tissues as well as nodular hyperplasia tissues.

Our study demonstrated pathophysiological relevance of HIF-1α and HIF-2α proteins in thyroid lesions, however the studied groups characterized wide variations in both subunits level. Similar observation was made by Burrows et al. [29], therefore the authors suggested regulation of HIF proteins via a combination of tumor genotype and microenvironment. We have observed significant positive correlation between *HIF1A* and *HIF2A* expression which may suggest similar regulation of both genes in the thyroid. Our results showed that HIFs mRNAs expression did not correlate with HIF proteins levels (Fig. 3b and c) which is not surprising taking into account posttranslational regulation of HIFs. It is well known that HIF-α proteins are controlled by 2-oxoglutarate dependent dioxygenases that require intracellular ascorbate for optimal activity [11, 12]. Several in vitro studies showed that ascorbate can suppress HIF-α protein stability and transcriptional activity [32–34]. Our in vitro studies also demonstrated a dose-dependent decrease in the expression of HIF-1α and GLUT1 in thyroid cancer cells after vitamin C treatment (Fig.4). This effect was associated with ascorbate uptake into treated cells. We have analyzed, for the first time, vitamin C content in thyroid samples and compared the obtained results with HIFs-α expression. Our results showed an inverse correlation between vitamin C content and HIF-1α level (*r* = −0.288, *p* = 0.025). The data suggests that high tumor tissue ascorbate level may decrease HIF-1 transcriptional activity in thyroid lesions. The ascorbate content of tumor tissue has been measured in some earlier studies with variable results. Brain and colorectal tumors contained significantly less ascorbate than normal tissue, whereas oral, lung and breast cancers had significantly more ascorbate than corresponding normal tissues [35–39]. More recent studies showed that endometrial and colorectal tumors of high histological grade had less ascorbate than matched, adjacent normal tissue [40–42]. In our study, we did not observe any differences in vitamin C levels between thyroid lesions. However, in contrast to previously mentioned studies we did not compare the ascorbate level between thyroid lesions and normal tissue. The results showing the association between HIF-1 level and ascorbate content in thyroid lesions are in accordance with studies concerning other tumors. Kuiper et al. [40] showed that endometrial tumors with the highest HIF-1α protein content were ascorbate deficient. The authors showed the same results in case of colorectal cancer [41]. They found a strong inverse relationship between the HIF-1 pathway score and tumor ascorbate content. Protein levels of VEGF and BNIP3 that are HIF-1-controlled pro-survival target genes were inversely correlated to tumor ascorbate content [41]. Intracellular accumulation of ascorbate is mostly dependent on expression of the SVCT transporters. On the other hand it has been suggested that ascorbate is oxidized in the extracellular environment and then GLUT transporters mediate DHAA uptake into the tumors [43, 44]. Our previous data suggested that GLUT1 was the main DHAA transporter in thyroid cancer cells [23]. Intracellular DHAA is rapidly reduced to ascorbate by a range of biological reducers and enzyme systems [42]. In the present study, we have shown that treatment of thyroid cancer cells with vitamin C impacts the amount of GLUT1 protein. There was also a tendency towards inverse association between GLUT1 expression and vitamin C content in thyroid tissue samples. Similarly, Kuiper et al. [41] showed an association between ascorbate content and GLUT1 expression in colorectal cancer. The ascorbate ratio (tumor: normal) was inversely correlated to GLUT-1 protein level.

## Conclusions

Results found in this study demonstrate that HIF-1α activation is associated with vitamin C content in thyroid lesions. Our findings revealed that high tumor tissue ascorbate level could limit the expression of HIF-1α and its targets in thyroid lesions. Therefore, a better understanding of ascorbate pharmacokinetics and mechanism of action in tumors may improve vitamin C clinical trials.

## Abbreviations

AA: Ascorbate
ARNT: Aryl hydrocarbon receptor nuclear translocator protein
bHLH: Basic helix-loop-helix
DHAA: Dehydroascorbic acid
DTT: Dithiothreitol
FA: Follicular adenoma
FC: Follicular carcinoma
GLUT: Glucose transporter
HIF: Hypoxia inducible factor
HPLC: High-performance liquid chromatography
HRE: Hypoxia response element
IOD: Integrated optical density
NG: Nodular goiter
PC: Papillary carcinoma
PER: Period circadian protein
PHDs: Prolyl hydroxylases
qRT-PCR: Quantitative real-time polymerase chain reaction
SIM`: Single-minded protein
SLC2A1/3: Solute carrier Family 2 Member 1/3, glucose transporter genes
SVCTs: Na + − dependent vitamin C transporters
TAA: Total amount of vitamin C
